# Interleukin-12p35 deficiency enhances mitochondrial dysfunction and aggravates cardiac remodeling in aging mice

**DOI:** 10.18632/aging.102609

**Published:** 2020-01-04

**Authors:** Jing Ye, Yuan Wang, Zhen Wang, Ling Liu, Zicong Yang, Di Ye, Menglong Wang, Yao Xu, Jishou Zhang, Mengmeng Zhao, Jianfang Liu, Yingzhong Lin, Qingwei Ji, Jun Wan

**Affiliations:** 1 Department of Cardiology, Renmin Hospital of Wuhan University, Cardiovascular Research Institute, Wuhan University, Hubei Key Laboratory of Cardiology, Wuhan 430060, China; 2 Department of Thyroid Breast Surgery, Renmin Hospital of Wuhan University, Wuhan 430060, China; 3 Emergency and Critical Care Center, Beijing Anzhen Hospital, Capital Medical University, Beijing Institute of Heart, Lung, and Blood Vessel Diseases, and Beijing Lab for Cardiovascular Precision Medicine, Beijing 100029, China; 4 Department of Cardiology, The People’s Hospital of Guangxi Zhuang Autonomous Region, Nanning 530021, China

**Keywords:** aging, cardiac remodeling, mitochondrial dysfunction, apoptosis-inducing factor, cardiomyocyte apoptosis

## Abstract

Our previous studies have demonstrated that interleukin-12p35 knockout (IL-12p35 KO) regulates the progression of various cardiovascular diseases, such as acute cardiac injury and hypertension. The aims of this study were to investigate whether IL-12p35 KO affects aging-related cardiac remodeling and to explore the possible mechanisms. First, the effects of IL-12p35 KO on heart structure and function were detected, and the results showed that IL-12p35 KO exacerbated cardiac remodeling and increased cardiac senescence-related protein levels in aged mice. In addition, whether IL-12p35 KO regulates cardiac senescence-related protein expression, cardiac mitochondrial dysfunction and cardiomyocyte apoptosis was also investigated. IL-12p35 KO increased mitochondrial calcium fluorescence intensity and ROS fluorescence intensity, while it reduced mitochondrial membrane potential. Furthermore, reduced mitochondrial complex (I-IV) activity and ATP levels and increased apoptosis-inducing factor (AIF)-related cardiomyocyte apoptosis were observed in aged IL-12p35 KO mice compared with wild-type mice. Our results demonstrate that aging is aggravated by IL-12p35 KO and that the mechanism may be related to exacerbation of mitochondrial dysfunction and AIF-related cardiomyocyte apoptosis.

## INTRODUCTION

Cardiac remodeling is an essential process associated with chronic heart failure, and inhibiting or delaying the development of such remodeling can significantly decrease the occurrence and improve the prognosis of chronic heart failure [1, 2]. The factors involved in cardiac remodeling are extremely complex, but pathological factors, including inflammatory responses, oxidative stress, apoptosis and autophagy, have been proven to be involved in the occurrence and progression of the process [3, 4]. Increasing evidence has demonstrated that aging is also associated with cardiac remodeling [5, 6].

Interleukins (ILs) are a group of cytokines that perform different functions, several of which have been proven to be closely related to aging. In clinical experiments, the expression levels of both IL-1β and IL-6 have been found to be significantly elevated in aging brains [7]. The levels of both IL-7 and IL-7 receptors gradually decrease with age, and in the aging population, lower 10-year all-cause mortality rates have been observed in individuals with higher IL-7 levels than in those with lower IL-7 levels [8]. In an animal study, overexpression of IL-10 in smooth muscle was found to significantly reduce inflammatory responses, ameliorate insulin resistance and accelerate glucose metabolism in aging mice [9]. In addition, in a mouse model of aging-associated perioperative neurocognitive disorders, administration of IL-17A significantly reduced inflammatory responses, disrupted the blood-brain barrier and improved cognitive function, while the opposite biological effects were observed upon administration of an IL-17A-neutralizing antibody [10].

IL-12p35 is a common component of IL-12 and IL-35, and IL-12p35 knockout (KO) is closely related to cardiovascular diseases. We and other researchers have found that IL-12p35 KO is involved in a variety of cardiovascular diseases [11–15]. In an early study, IL-12p35 KO was reported to aggravate angiotensin II-induced cardiac fibrosis [11]. Furthermore, another study demonstrated that IL-12p35 deficiency improved cardiac repair after myocardial infarction [12]. Our studies found that IL-12p35 deletion aggravated acute cardiac injury in doxorubicin-treated mice and elevated blood pressure in angiotensin II-infused mice [13, 14]. A recent study found that atherosclerosis induced by a high-fat diet could be aggravated by IL-12p35 KO in apolipoprotein KO mice [15]. Considering the important role of IL-12p35 KO in the process of cardiovascular diseases and the close correlation between IL-12p35 KO and aging, we speculated that IL-12p35 KO may be involved in cardiac aging. In the present study, we identified the role of IL-12p35 KO in aging-related cardiac remodeling and attempted to elucidate the possible mechanisms.

## RESULTS

### IL-12p35 KO exacerbates cardiac dysfunction in aging mice

**Figure 1:**
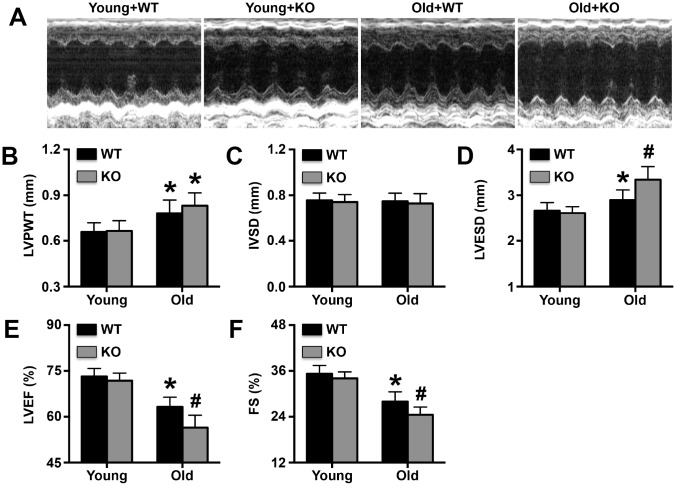
**Effects of IL-12p35 KO on cardiac function.** (**A**). Representative M-mode echocardiograms, (**B**). LVPWT, (**C**). IVSD, (**D**). LVESD, (**E**). LVEF and (**F**). FS for each group. * p<0.05 vs. the young WT group; ^#^ p<0.05 vs. the aged WT group; n=11-12 for each group.

By the end of the 25^th^ month, 18 aged WT mice had died, and 52 aged IL-12p35 KO mice had died; none of the young WT mice and young IL-12p35 KO mice died. IL-12p35 KO significantly affected cardiac function in aging mice (Figure 1A). In addition, old mice exhibited higher LVPWT and IL-12p35 KO did not affect LVPWT in old mice (Figure 1B). No differences in IVSD were observed among the 4 groups (Figure 1C). Moreover, IL-12p35 KO further increased LVESD while decreasing LVEF and FS in aged mice (Figure 1D–1F).

### IL-12p35 KO aggravates aging-related cardiac remodeling

**Figure 2:**
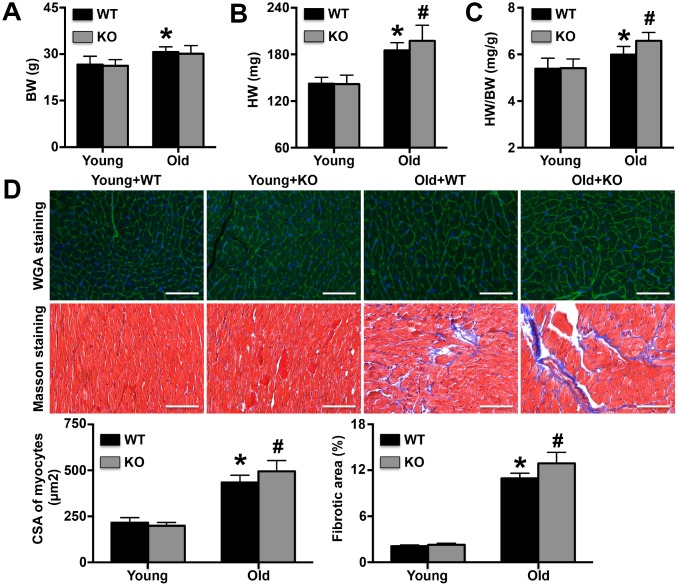
**Regulatory role of IL-12p35 KO in cardiac remodeling.** (**A**–**C**). Body weights (BW), heart weights (HW) and HW/BW ratios were measured in the four groups; n=10 for each group. (**D**). The cross-sectional areas (CSA) of cardiomyocytes and cardiac fibrotic areas were measured by WGA staining and Masson staining, respectively (200x). * p<0.05 vs. the young WT group; ^#^ p<0.05 vs. the aged WT group; n=5 for each group.

No significant changes in body weight were observed upon IL-12p35 KO (Figure 2A). However, in IL-12p35 KO mice, the heart was heavier, and the ratio of heart weight to body weight was increased (Figure 2B and 2C). Cardiomyocyte cross-sectional area and cardiac fibrosis showed trends similar to those of the heart weights or heart weight-to-body weight ratios (Figure 2D).

### IL-12p35 KO increases the expression of senescence-related proteins

**Figure 3:**
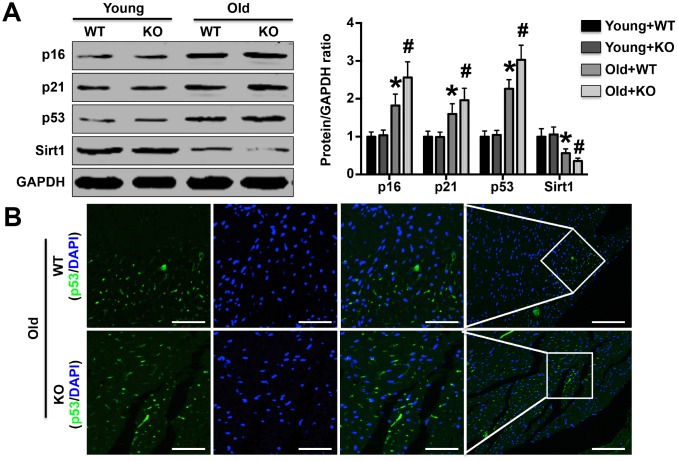
**Effects of IL-12p35 KO on the expression of aging marker-related proteins.** (**A**) The left ventricular p16, p21, p53 and Sirt1 expression levels in each group were investigated by Western blotting analysis; n=10-11 for each group. (**B**) Cardiac p53 expression was detected by immunofluorescence staining (200x); n=5 for each group. * p<0.05 vs. the young WT group; ^#^ p<0.05 vs. the aged WT group.

As shown by Western blotting analysis, p16, p21 and p53 expression was significantly increased by IL-12p35 KO (Figure 3A). In contrast, Sirt1 levels were noticeably decreased in aged IL-12p35 KO mice compared with aged WT mice (Figure 3A). Immunofluorescence staining also showed that IL-12p35 KO elevated the expression of p53 in aging mice (Figure 3B).

### IL-12p35 KO increases mitochondrial calcium and ROS fluorescence intensity but decreases mitochondrial membrane potential

**Figure 4:**
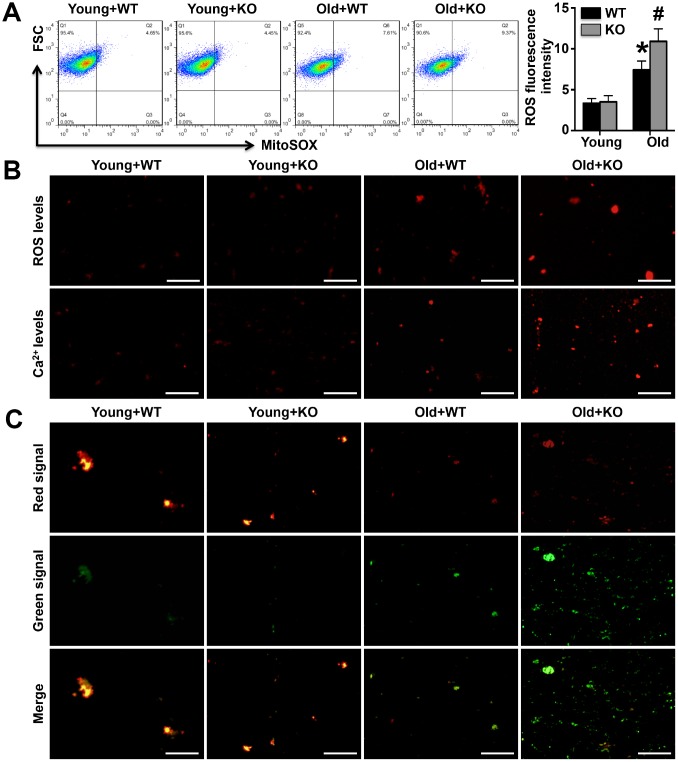
**Mitochondrial fluorescence intensity of calcium and ROS and membrane potentials were detected.** (**A**). The mitochondrial ROS fluorescence intensity was investigated by the Flow Cytometry analyses. (**B**). The MitoSOX Red Mitochondrial Superoxide Indicator probe and the Rhod-2 AM probe were used to mark mitochondrial calcium ions and ROS, respectively (400x). (**C**). The mitochondrial membrane potential was detected using the JC-1 probe (400x). N=5-6 for each group.

As the results of Flow Cytometry analyses, ROS intensity was increased in Old WT mice and further elevated in Old KO mice (Figure 4A). As shown by mitochondrial staining, the fluorescence signal intensities of mitochondrial calcium and ROS were significantly elevated in aged WT mice compared to young WT mice and were further increased in aged IL-12p35 KO mice compared to aged WT mice; however, IL-12p35 KO did not affect the mitochondrial calcium or ROS fluorescence signal intensities in young mice (Figure 4B). In young WT and young IL-12p35 KO mice, the JC-1 probe mainly produced a red-orange fluorescence signal, and the green signal was barely visible; in contrast, a strong green fluorescence signal was observed in aged WT mice that was further enhanced upon IL-12p35 KO in aged mice (Figure 4C).

### IL-12p35 KO exacerbates mitochondrial dysfunction in aging mice

**Figure 5:**
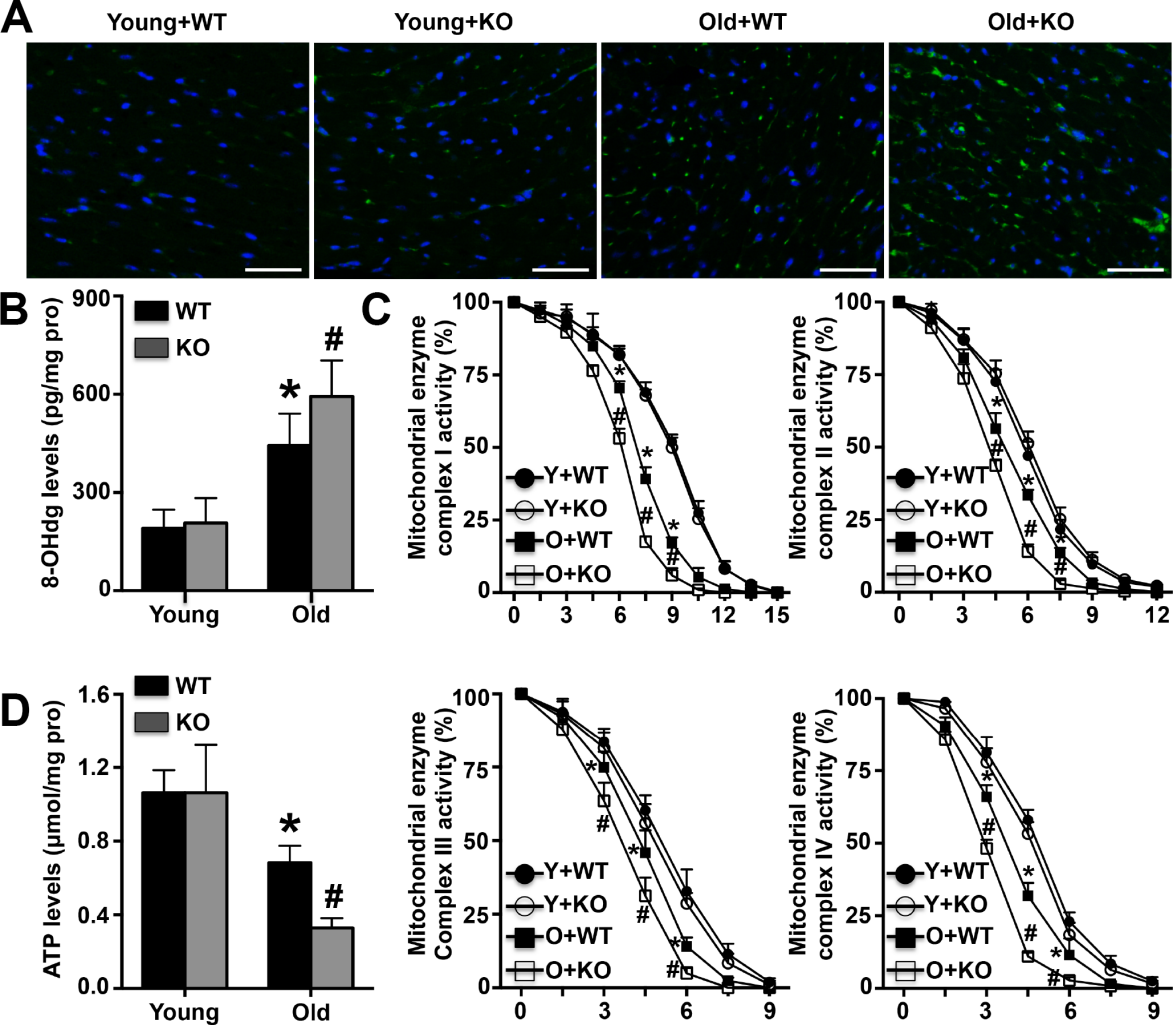
**Effects of IL-12p35 KO on mitochondrial dysfunction.** (**A**, **B**). Cardiac 8-OHdg expression (200x) and mitochondrial 8-OHdg levels were detected. (**C**). The activity of mitochondrial enzyme complexes I, II, III and IV in the four groups was measured. (**D**). The mitochondrial ATP levels in each group were detected. * p<0.05 vs. the young WT group; ^#^ p<0.05 vs. the aged WT group; n=4-5 for each group.

Both cardiac 8-OHdg expression and cardiac mitochondrial 8-OHdg levels were measured, and the results showed that senescence-mediated increases in 8-OHdg were further amplified by IL-12p35 KO in both hearts and cardiac mitochondria (Figure 5A and 5B). Among aging mice, the activity of the cardiac mitochondrial enzyme complexes I, II, III, and IV decreased more rapidly in IL-12p35 KO mice than in WT mice (Figure 5C). Similarly, senescence-mediated reductions in ATP levels in cardiac mitochondria were also exacerbated by IL-12p35 KO (Figure 5D).

### IL-12p35 KO aggravates AIF-related apoptosis in aging mice

**Figure 6:**
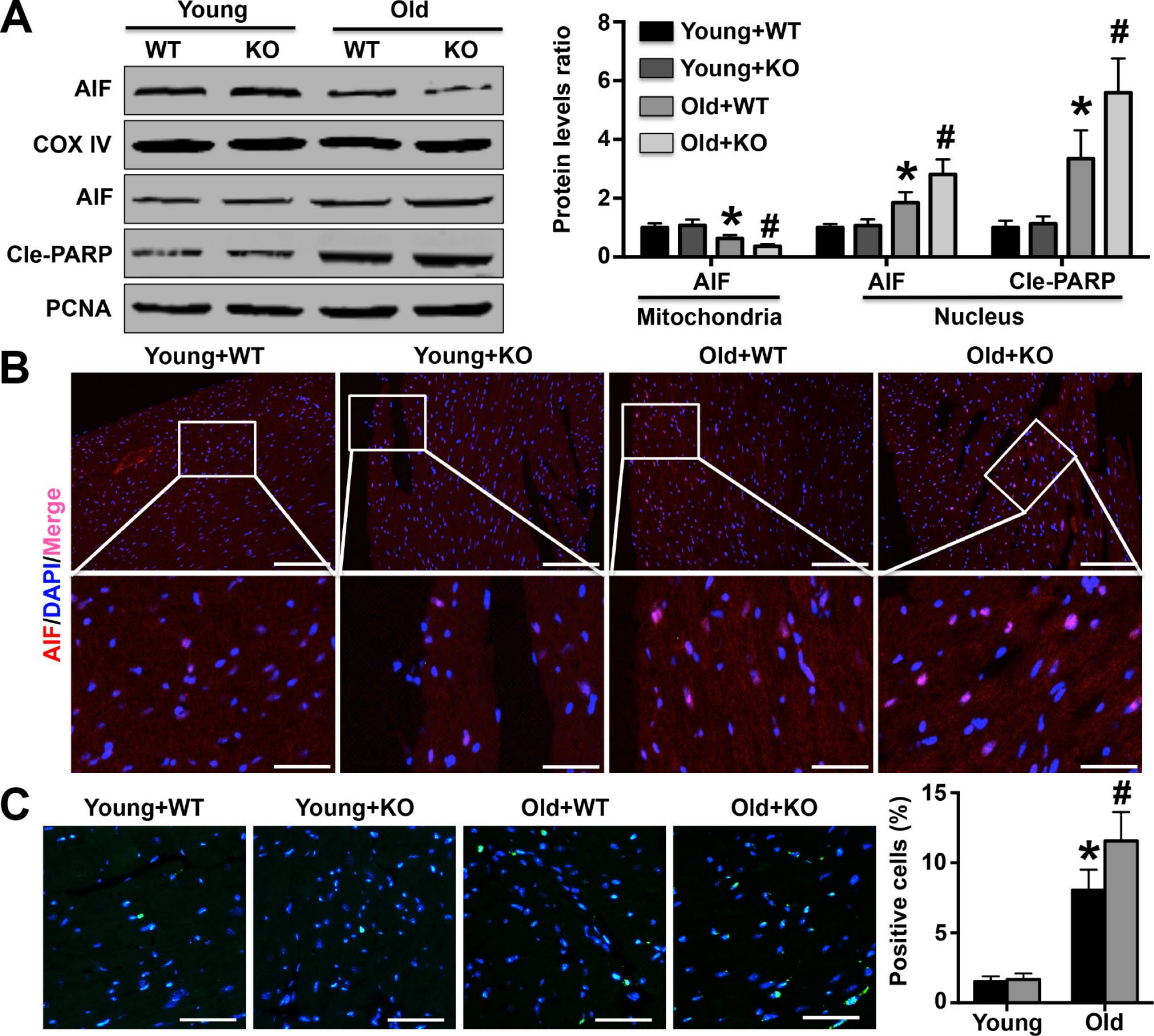
**Effects of IL-12p35 KO on cardiomyocyte apoptosis.** (**A**) Mitochondrial AIF expression and nuclear AIF and Cle-PARP expression were measured by Western blotting analysis. (**B**) Cardiac AIF expression was detected by immunofluorescence staining (200x). (**C**) TUNEL staining was performed to mark the apoptotic cells and the positive cell numbers in each group (200x). * p<0.05 vs. the young WT group; ^#^ p<0.05 vs. the aged WT group; n=5 for each group.

Compared with young WT mice, cardiac mitochondrial AIF expression was decreased in aged WT mice and further decreased in aged IL-12p35 KO mice (Figure 6A). However, aging mice showed higher AIF expression in cardiomyocyte nuclei, and AIF expression was further increased by IL-12p35 KO (Figure 6A). Cle-PARP expression in cardiomyocyte nuclei showed trends similar to those of AIF levels in cardiomyocyte nuclei (Figure 6A). The results of immunofluorescence staining also showed that the expression of AIF in cardiomyocyte nuclei was significantly increased in aging IL-12p35 KO mice (Figure 6B). IL-12p35 KO also significantly increased apoptosis of cardiomyocytes in aging mice (Figure 6C).

## DISCUSSION

In this study, we found, for the first time, that aging IL-12p35 KO mice show poorer cardiac function and more severe myocardial remodeling than aging WT mice. In addition, cardiac mitochondrial dysfunction in IL-12p35 KO mice was exacerbated, as demonstrated by increased calcium ion levels, decreased membrane potential levels, decreased enzyme complex activity and significantly reduced ATP synthesis. A number of manifestations of cardiac mitochondrial dysfunction can lead to increased levels of AIF in cardiac nuclei, leading to cardiomyocyte apoptosis. These results suggest that IL-12p35 KO causes cardiac mitochondrial dysfunction, which leads to cardiomyocyte apoptosis and further aggravates cardiac remodeling related to aging.

Cardiac remodeling has been regarded as a typical age-related disease in recent years [16]. As individuals age, the heart ages gradually in a complex pathological process and can display a range of common physiological manifestations and morphological feature alterations, including structural changes, reduced reserve and large deposits of collagen fibers [17, 18]. In addition, some specific physiological changes have also been found in aging hearts, including increases in the expression of various apoptosis markers [6, 19]. Thus far, seven members of the Sirtuin family have been identified as anti-aging proteins. Among them, Sirt1 has been reported to significantly delay the progression of cardiac aging and to inhibit the deterioration of cardiac dysfunction in aging animals [20]. Therefore, in addition to detecting cardiac structure and function and the protein expression of cardiac aging markers, we also detected sirt1 expression. Our results showed that after IL-12p35 KO, cardiac dysfunction was further deteriorated, cardiac remodeling was more severe, and the expression levels of p16, p21 and p53 were further increased in aging mice, while the expression of sirt1 was further decreased. These results suggest that IL-12p35 KO exacerbates aging-related cardiac remodeling and that these effects may be associated with aggravation of aging. Our research and that of other researchers fully illustrated that the process of aging does not simply move forward with time but is also regulated by a variety of aging-related genes or an imbalance in the expression of anti-aging and pro-aging genes.

Research on mitochondria and age-related diseases has been ongoing for more than 40 years, and data from animal studies and clinical trials suggest that impairment of mitochondrial function can promote the development of a variety of age-related diseases, including cardiac aging [21–23]. Replication errors caused by mutations in mitochondrial DNA were initially thought to be the most important causes of aging; however, recently, increasing evidence has shown that oxidative stress injury and the resulting high calcium ion levels leading to mitochondrial DNA damage and subsequent declines in membrane potential are the most important fundamental causes [21–24]. In addition, reductions in abnormal oxidative stress levels in mitochondria can significantly improve mitochondrial damage and dysfunction and inhibit the development of aging [21–24]. To investigate the mechanism by which IL-12p35 KO aggravates cardiac remodeling, mitochondrial ROS levels, calcium ion levels and membrane potentials were detected. The results showed that ROS expression and calcium ion levels in cardiac mitochondria were significantly increased after IL-12p35 KO in aged mice, and the green signal was further enhanced by JC-1 staining. These results indicate that further deterioration of mitochondrial dysfunction is one of the important mechanisms by which IL-12p35 KO promotes aging and aggravates cardiac remodeling. Additionally, the worsening of mitochondrial dysfunction may be accompanied by increases in oxidative stress levels, calcium ion concentrations and concomitant decreases in membrane potential. Mitochondrial dysfunction or mitochondrial injury caused by a variety of pathological factors are crucial to the progression of aging. Reducing the pathological factors related to mitochondria or protecting against mitochondrial dysfunction may be beneficial to delaying sunburn or alleviating age-related diseases.

ATP is an unstable high-energy phosphoric acid compound and is also the most direct and important energy substance in organisms. More than 90% of ATP in organisms is produced by the mitochondrial inner membrane. A variety of pathological factors, including enhanced inflammatory responses and oxidative stress injury, can significantly affect ATP production by this membrane, and ATP synthesis abnormalities or reductions can lead to many serious diseases, including fatty liver, abnormal glucose metabolism and Alzheimer's disease [25, 26]. The heart is one of the organs with the highest mitochondrial content, and reduced ATP synthesis due to abnormal mitochondrial function has been found in a variety of heart-related diseases, although abnormal ATP production mediated by mitochondrial dysfunction has not been found to be involved in cardiac aging. In cases in which mitochondrial dysfunction was confirmed in aging hearts, we further examined the production of mitochondrial ATP. We found that mitochondrial ATP synthesis decreased in aging hearts and further decreased after IL-12p35 KO. These results suggest that IL-12 KO aggravates myocardial remodeling and may be associated with reduced ATP production by mitochondria in aging hearts. Because redox reactions mediated by mitochondrial enzyme complexes, also known as mitochondrial oxidative respiratory chains, are essential for ATP production, we also examined the activity of mitochondrial enzyme complexes I, II, III and IV, and the results showed that IL-12p35 KO further inhibited the activity of these complexes in aging mice. These results further support our conclusion that a further reduction in ATP synthesis caused by aggravation of mitochondrial dysfunction may be an important reason for the exacerbation of myocardial remodeling and cardiac dysfunction caused by IL-12p35 KO. Our study is the first to show that energy failure induced by mitochondrial dysfunction is involved in the aging process, which is regulated by IL-12p35 KO. These results further illustrate the complexity of the aging process, which is not caused by any one factor but is a common result of multiple pathological factors or injuries.

AIF is the first factor that can induce caspase-independent apoptosis and exists in the mitochondrial inner membrane [27]. During the development of apoptosis, AIF translocates from mitochondria to the cytoplasm and then into the nucleus, and increased AIF in the nucleus has been shown to increase cardiomyocyte apoptosis in aging hearts [19]. Our results suggest that IL-12p35 KO promotes mitochondrial AIF translocation to the nucleus. In contrast to AIF, PARP is mainly a DNA repair enzyme existing in the nucleus, and activated PARP can prevent DNA damage and inhibit apoptosis [28]. In a recent study, expression of activated PARP was also observed to increase in aging brain tissue, which was consistent with our results and closely related to both DNA damage and apoptosis of brain cells [29]. Moreover, activated PARP, which is an anti-apoptotic protein, is increased in aging heart and brain. One possible reason is that elevated cleaved PARP1 positive feedback can protect against aging-related heart and brain injury. In TUNEL staining, the trends in the numbers of positive cells were similar to the trends in AIF expression in nuclei; these findings could further suggest that IL-12p35 KO aggravates AIF-related cardiomyocyte apoptosis, which may be the mechanism underlying the exacerbation of cardiac remodeling. The essence of organ damage and dysfunction caused by aging may be that the role of injury factors exceeds that of protective factors in aging, leading to excessive apoptosis of tissue and organ cells.

Collectively, our findings suggest that IL-12p35 KO in mice aggravates aging-associated cardiac structural changes and functional impairment. Our data suggest that the injury-aggravating role of IL-12p35 deficiency in cardiac aging is attributable to the up-regulation of aging-associated caspase-independent cardiac apoptosis and therefore to the worsening of cardiac dysfunction. The results of this study may be especially important given the inevitability of aging.

## MATERIALS AND METHODS

### Experimental animals

Both wild-type (WT) and IL-12p35 KO mice bred on a C57BL/6 background were purchased from Jackson Laboratories. All mice were housed in a pathogen-free mouse room at Renmin Hospital of Wuhan University and fed a normal diet. Subsets of the WT mice (n=38) and IL-12p35 KO mice (n=72) aged 25 months were considered aged mice, while mice aged 3-5 months were used as controls (n=20 for each of these two groups). The study was approved by the ethics committee of Renmin Hospital of Wuhan University.

### Examination of cardiac structure and function

After the mice were anesthetized, cardiac ultrasound was performed by the laboratory technicians to examine the structure and function of the left ventricle (LV). The left ventricular posterior wall thickness (LVPWT), end-diastolic interventricular septal thickness (IVSD), left ventricular end-systolic diameter (LVESD), left ventricular end-diastolic diameter (LVEDD) and left ventricular ejection fraction (LVEF) data were recorded and analyzed. Fractional shortening (FS) was calculated according to the methods in previous reports.

### Histological analysis of the heart

After isolation, the hearts were fixed, dehydrated and sectioned. Wheat germ agglutinin (WGA) staining was performed to detect the cardiomyocyte cross-sectional area, and more than 100 cells were counted in each group. Masson staining was used to determine the cardiac fibrotic area. In addition, an anti-p53 antibody (GeneTex), an anti-8-hydroxy-2’-deoxyguanosine (8-OHdg) antibody (Santa Cruz) and an anti-apoptosis-inducing factor (AIF, GeneTex) antibody were used to detect cardiac p53, 8-OHdg and AIF expression, respectively. Finally, apoptotic cardiomyocytes were detected using a terminal deoxynucleotidyl transferase-mediated dUTP nick end labeling (TUNEL) kit (Sigma).

### Flow cytometry analyses

The separated fresh left ventricular tissue was immediately cut into small pieces and digested into individual cardiac cells. After centrifugation, the cells were obtained and implanted on the 6-well plates, and cardiomyocytes were obtained after the fibroblasts were removed by differential adherent method. Cardiomyocytes were collected and incubated with 0.5μM MitoSOX in dark for 30 minutes as previous study [30], and the ROS intensity was detected on the Flow Cytometer.

### Separation of mitochondria and nuclei

Mitochondria were isolated using a mitochondrial isolation kit according to the manufacturer's instructions (Cayman). Briefly, mitochondrial separation solution was added to fresh LV tissue, after which the tissue was centrifuged at 700 × *g* for 10 minutes. The supernatant was then collected and centrifuged at 6000 × *g* for 10 minutes. The supernatant was discarded, and the pellet was collected after washing twice to obtain the purified mitochondria.

The nuclei were obtained from LV tissue using a nuclear separation kit (Njjcbio). First, the remaining fresh LV tissue was lysed with the kit-provided lysis buffer, and the tissue homogenate was collected and centrifuged at 1000 × *g* for 10 minutes. The collected pellet contained the nuclei. Nuclei with high purity were obtained by washing the pellet twice with the kit washing solution.

### Detection of calcium ion levels, ROS levels and membrane potential in mitochondria

A portion of the purified mitochondria was resuspended using mitochondrial preservation solution. A Rhod-2 AM probe, a MitoSOX Red Mitochondrial Superoxide Indicator probe and a JC-1 probe (all from Cayman) were separately incubated with mitochondria in the dark for 30 minutes. After washing 3 times, the mitochondria were resuspended in 30 μl of mitochondrial preservation solution and smeared on slides before being detected by fluorescence microscopy.

### Detection of protein expression levels

Mitochondria and nuclei purified as described above as well as LV tissue were treated with RIPA lysis buffer for total protein separation. The protein from the different sources was quantified, and p16, p21, p53, and sirt1 expression in LV tissue; AIF and COX IV expression in mitochondria; and AIF, cleaved-PARP (Cle-PARP) and PCNA expression in nuclei were measured using Western blotting analysis as described in our previous studies [13, 14, 31].

### Detection of 8-OHdg levels, enzyme complex I-IV activation and ATP levels in mitochondria

The activity of mitochondrial enzyme complexes I-IV was measured according to the instructions provided by the kit manufacturer. Optical density (OD) values were read at appropriate wavelengths every 90 seconds to enable calculation of dynamic changes in the activity of each mitochondrial enzyme. The 8-OHdg levels and ATP levels in mitochondrial homogenates were detected based on the protocol, but the results are expressed as the ratios of the 8-OHdg and ATP levels to the total mitochondrial protein concentration.

### Data analysis

All data in this study are expressed as the mean ± standard deviation. There were more than three groups for all the data in this study; therefore, analysis of variance (ANOVA) followed by Tukey’s post hoc multiple comparisons test was performed to calculate differences in means among different groups. The null hypothesis was rejected when p < 0.05.

## References

[1] van Berlo JH, Maillet M, Molkentin JD. Signaling effectors underlying pathologic growth and remodeling of the heart. J Clin Invest. 2013; 123:37–45. 10.1172/JCI62839 23281408 PMC3533272

[2] Gjesdal O, Bluemke DA, Lima JA. Cardiac remodeling at the population level—risk factors, screening, and outcomes. Nat Rev Cardiol. 2011; 8:673–85. 10.1038/nrcardio.2011.154 22027657

[3] Kaplan A, Abidi E, Ghali R, Booz GW, Kobeissy F, Zouein FA. Functional, cellular, and molecular remodeling of the heart under influence of oxidative cigarette tobacco smoke. Oxid Med Cell Longev. 2017; 2017:3759186. 10.1155/2017/3759186 28808498 PMC5541812

[4] Mann DL, Bogaev R, Buckberg GD. Cardiac remodelling and myocardial recovery: lost in translation? Eur J Heart Fail. 2010; 12:789–96. 10.1093/eurjhf/hfq113 20675667

[5] Hua Y, Zhang Y, Ceylan-Isik AF, Wold LE, Nunn JM, Ren J. Chronic Akt activation accentuates aging-induced cardiac hypertrophy and myocardial contractile dysfunction: role of autophagy. Basic Res Cardiol. 2011; 106:1173–91. 10.1007/s00395-011-0222-8 21901288

[6] Inuzuka Y, Okuda J, Kawashima T, Kato T, Niizuma S, Tamaki Y, Iwanaga Y, Yoshida Y, Kosugi R, Watanabe-Maeda K, Machida Y, Tsuji S, Aburatani H, et al. Suppression of phosphoinositide 3-kinase prevents cardiac aging in mice. Circulation. 2009; 120:1695–703. 10.1161/CIRCULATIONAHA.109.871137 19822807

[7] Shen F, Jiang L, Han F, Degos V, Chen S, Su H. Increased inflammatory response in old mice is associated with more severe neuronal injury at the acute stage of ischemic stroke. Aging Dis. 2019; 10:12–22. 30705764 10.14336/AD.2018.0205 PMC6345332

[8] Nguyen V, Mendelsohn A, Larrick JW. Interleukin-7 and Immunosenescence. J Immunol Res. 2017; 2017:4807853. 10.1155/2017/4807853 28484723 PMC5397725

[9] Dagdeviren S, Jung DY, Friedline RH, Noh HL, Kim JH, Patel PR, Tsitsilianos N, Inashima K, Tran DA, Hu X, Loubato MM, Craige SM, Kwon JY, et al. IL-10 prevents aging-associated inflammation and insulin resistance in skeletal muscle. FASEB J. 2017; 31:701–10. 10.1096/fj.201600832R 27811060 PMC5240661

[10] Ni P, Dong H, Wang Y, Zhou Q, Xu M, Qian Y, Sun J. IL-17A contributes to perioperative neurocognitive disorders through blood-brain barrier disruption in aged mice. J Neuroinflammation. 2018; 15:332. 10.1186/s12974-018-1374-3 30501622 PMC6267879

[11] Li Y, Zhang C, Wu Y, Han Y, Cui W, Jia L, Cai L, Cheng J, Li H, Du J. Interleukin-12p35 deletion promotes CD4 T-cell-dependent macrophage differentiation and enhances angiotensin II-Induced cardiac fibrosis. Arterioscler Thromb Vasc Biol. 2012; 32:1662–74. 10.1161/ATVBAHA.112.249706 22556333

[12] Kan X, Wu Y, Ma Y, Zhang C, Li P, Wu L, Zhang S, Li Y, Du J. Deficiency of IL-12p35 improves cardiac repair after myocardial infarction by promoting angiogenesis. Cardiovasc Res. 2016; 109:249–59. 10.1093/cvr/cvv255 26614777

[13] Ye J, Huang Y, Que B, Chang C, Liu W, Hu H, Liu L, Shi Y, Wang Y, Wang M, Zeng T, Zhen W, Xu Y, et al. Interleukin-12p35 knock out aggravates doxorubicin-induced cardiac injury and dysfunction by aggravating the inflammatory response, oxidative stress, apoptosis and autophagy in mice. EBioMedicine. 2018; 35:29–39. 10.1016/j.ebiom.2018.06.009 30228093 PMC6154773

[14] Ye J, Que B, Huang Y, Lin Y, Chen J, Liu L, Shi Y, Wang Y, Wang M, Zeng T, Wang Z, Hu H, Xu Y, et al. Interleukin-12p35 knockout promotes macrophage differentiation, aggravates vascular dysfunction, and elevates blood pressure in angiotensin II-infused mice. Cardiovasc Res. 2019; 115:1102–13. 10.1093/cvr/cvy263 30395167

[15] Huang Y, Hu H, Liu L, Ye J, Wang Z, Que B, Liu W, Shi Y, Zeng T, Shi L, Ji Q, Chang C, Lin Y. Interleukin-12p35 deficiency reverses the Th1/Th2 imbalance, aggravates the Th17/Treg imbalance, and ameliorates atherosclerosis in ApoE-/- mice. Mediators Inflamm. 2019; 2019:3152040. 10.1155/2019/3152040 31093011 PMC6481022

[16] Lakatta EG, Levy D. Arterial and cardiac aging: major shareholders in cardiovascular disease enterprises: Part II: the aging heart in health: links to heart disease. Circulation. 2003; 107:346–54. 10.1161/01.CIR.0000048893.62841.F7 12538439

[17] Horn MA, Trafford AW. Aging and the cardiac collagen matrix: novel mediators of fibrotic remodelling. J Mol Cell Cardiol. 2016; 93:175–85. 10.1016/j.yjmcc.2015.11.005 26578393 PMC4945757

[18] Shirakabe A, Ikeda Y, Sciarretta S, Zablocki DK, Sadoshima J. Aging and autophagy in the heart. Circ Res. 2016; 118:1563–76. 10.1161/CIRCRESAHA.116.307474 27174950 PMC4869999

[19] Hua Y, Robinson TJ, Cao Y, Shi GP, Ren J, Nair S. Cathepsin K knockout alleviates aging-induced cardiac dysfunction. Aging Cell. 2015; 14:345–51. 10.1111/acel.12276 25692548 PMC4406663

[20] Yuan Y, Cruzat VF, Newsholme P, Cheng J, Chen Y, Lu Y. Regulation of SIRT1 in aging: roles in mitochondrial function and biogenesis. Mech Ageing Dev. 2016; 155:10–21. 10.1016/j.mad.2016.02.003 26923269

[21] Kauppila TE, Kauppila JH, Larsson NG. Mammalian mitochondria and aging: an update. Cell Metab. 2017; 25:57–71. 10.1016/j.cmet.2016.09.017 28094012

[22] Zhang H, Menzies KJ, Auwerx J. The role of mitochondria in stem cell fate and aging. Development. 2018; 145:145. 10.1242/dev.143420 29654217 PMC5964648

[23] Todorova V, Blokland A. Mitochondria and synaptic plasticity in the mature and aging nervous system. Curr Neuropharmacol. 2017; 15:166–73. 10.2174/1570159X14666160414111821 27075203 PMC5327446

[24] Mora AL, Bueno M, Rojas M. Mitochondria in the spotlight of aging and idiopathic pulmonary fibrosis. J Clin Invest. 2017; 127:405–14. 10.1172/JCI87440 28145905 PMC5272191

[25] Softic S, Cohen DE, Kahn CR. Role of dietary fructose and hepatic de novo lipogenesis in fatty liver disease. Dig Dis Sci. 2016; 61:1282–93. 10.1007/s10620-016-4054-0 26856717 PMC4838515

[26] Chen Z, Zhong C. Decoding Alzheimer’s disease from perturbed cerebral glucose metabolism: implications for diagnostic and therapeutic strategies. Prog Neurobiol. 2013; 108:21–43. 10.1016/j.pneurobio.2013.06.004 23850509

[27] Dyshlovoy SA, Rast S, Hauschild J, Otte K, Alsdorf WH, Madanchi R, Kalinin VI, Silchenko AS, Avilov SA, Dierlamm J, Honecker F, Stonik VA, Bokemeyer C, von Amsberg G. Frondoside A induces AIF-associated caspase-independent apoptosis in Burkitt lymphoma cells. Leuk Lymphoma. 2017; 58:2905–15. 10.1080/10428194.2017.1317091 28508718

[28] Shah AP, Patel CN, Sureja DK, Sanghavi KP. A review on DNA repair inhibition by PARP inhibitors in cancer therapy. Folia Med (Plovdiv). 2018; 60:39–47. 10.1515/folmed-2017-0067 29668451

[29] Jeong YJ, Son Y, Han NK, Choi HD, Pack JK, Kim N, Lee YS, Lee HJ. Impact of long-term RF-EMF on oxidative stress and neuroinflammation in aging brains of C57BL/6 mice. Int J Mol Sci. 2018; 19:19. 10.3390/ijms19072103 30029554 PMC6073444

[30] Kauffman ME, Kauffman MK, Traore K, Zhu H, Trush MA, Jia Z, Li YR. MitoSOX-Based Flow Cytometry for Detecting Mitochondrial ROS. React Oxyg Species (Apex). 2016; 2:361–70. 10.20455/ros.2016.865 29721549 PMC5926237

[31] Ye J, Ji Q, Liu J, Liu L, Huang Y, Shi Y, Shi L, Wang M, Liu M, Feng Y, Jiang H, Xu Y, Wang Z, et al. Interleukin-22 promotes blood pressure elevation and endothelial dysfunction in angiotensin II-treated mice. J Am Heart Assoc. 2017; 6:6. 10.1161/JAHA.117.005875 28974499 PMC5721831

